# Abortion, with Retention of Placenta for Five Months without Sepsis

**Published:** 1888-06

**Authors:** Elbert Wing

**Affiliations:** Demonstrator of Pathology at Chicago Medical College; 3266 Cottage Grove Avenue


### REPORT OF A CASE OF ABORTION IN THE THIRD MONTH OF GESTATION, WITH RETENTION OF THE PLACENTA FOR FIVE MONTHS WITHOUT SEPSIS.

BY ELBERT WING, A. M., M. D.,
Demonstrator of Pathology at Chicago Medical College.

One of the physicians in whose practice this case occurred is personally known to the writer, in whose opinion there is no reason to doubt the accuracy of the facts as reported. The history of the case, together with the size and nature of the tumor expelled from the uterus, clearly indicate its nature, and this was confirmed by microscopical examination of a small portion. This portion of the tumor, particularly described later in this report, was handed to me with a request for an examination without a knowledge of the case sufficient to even suggest its nature.

The woman was a patient of Drs. Green and Morse, of Shullsburg, Wisconsin, who furnish the clinical history of the case.

Mrs. M., 35 years old, married, has borne six children, and has had several miscarriages. Until the complications associated with this abortion, her menstruation had been normal. Six months prior to the time at which her last pregnancy began, the menstrual hæmorrhage increased notably in quantity, and at times continued almost during the entire month. She thinks conception occurred at the time when this menorrhagia spontaneously ceased, and her pregnancy was normal up to the third month, during which the abortion occurred and the menorrhagia began again. The patient assigns no cause for the abortion.

With a view of controlling the uterine hæmorrhage she was given ergot, but the preparation and dosage are not stated. This hæmorrhage continued for five months, when Dr. Green was consulted.

He " put the patient thoroughly under the influence of the fluid extract of ergot," and twenty-four hours later the tumor was expelled. It is described as " four inches long, one and one-half inches thick, in the shape of a roll, and white in color."

It is now three months since the tumor was expelled, and as far as her physicians know the patient is in good health.

The following description of the piece of the tumor given the writer for examination, was made at the time, and is given here as of interest in the absence of one of the entire mass, which latter is not available.

The piece of tissue has an average thickness of ten millimetres and is irregularly circular in outline. It appears as if cut from a semi-cylindrical mass. The larger cut-surface measures thirty-seven millimetres in its greatest, and thirty-four in its least, diameter. The corresponding measurements of the smaller cut-surface are respectively thirty-four and thirty millimetres.

The *cut-surface* presents a fibrous and granular appearance, and is of a pale pink color. This color is made up by a combination of numerous round white spots which vary from one to three millimetres in diameter, and larger areas, irregular in size and outline, which closely resemble hæmorrhagic spots. Around the edge of this surface there is a band of paler tissue which averages three millimetres in width, but is irregular in outline and has not the appearance of an investing membrane.

The *edge* presents many convolutions and sulci which vary in size aud shape. The sulci vary from mere lines to two and three millimetres in depth. The surface of the edge is slightly rough from the presence of very fine, short fibrous threads, and in general appearance it resembles the uterine surface of a compressed and hardened placenta.

The tissue is easily friable, and when broken reveals a fibrous, stringy, and irregular surface. The small white spots in the cut-surface prove to be the ends of white, round fibres or bands which run obliquely from one surface to the other. These bands are quite tough, somewhat elastic and compressible.

Small pieces of the tissues were imbedded in celloidin, and sections cut both parallel with, and perpendicular to, the cut-surface.

The tissue has undergone retrograde changes to such an extent that the nuclei take the staining reagents poorly, and are of all sizes and irregular in outline. The rest of the tissue is an almost structureless granular mass. Partly owing to irregular refraction of the light, and partly to the arrangement of the staining material used, an appearance is presented in some parts of the sections which strongly suggests the arborescent arrangement of the placental villi. This appearance is best seen in sections cut parallel with the cut-surface. In the other sections there are irregular groupings of spaces of varying shape and size, circular and oval, which resemble a section cut through a bunch of vessels of various sizes.

In both sets of sections the rest of the area is made up principally of granular matter which resembles destroyed blood corpuscles.

Charpentier (Wood's Library, 1887, Vol. II, p. 332) says, " These placenta thus retained for a longer or shorter time in the uterus, may: *a.* Be expelled not altered, not putrid. *b.* Altered and putrid. *c.* With symptons of septic fever. *d.* Without such symptoms. *e.* Absorbed. *f.* Remain indefinitely in the uterus." He quotes Cazeaux, who thinks that " the integrity of vascular connection gives them life, and explains the innocuousness of this prolonged retention." According to Charpentier " Schüller has noted retention for 11 weeks; Metz for two and a half months; Prost, 103 days; Plasse, 15 weeks; and Reichman, 13 weeks."

It is, of course, a matter of regret that the clinical history of this case is not more fully known, but it is nevertheless thought worthy of record.

3266 Cottage Grove Avenue.

